# Heerfordt-Waldenström Syndrome Manifesting as Cardiac Sarcoidosis

**DOI:** 10.7759/cureus.10619

**Published:** 2020-09-23

**Authors:** Sebastian Mikulic, Pujan Patel, Sandra Sheffield, Fadi Kandah, Gladys Velarde

**Affiliations:** 1 Internal Medicine, University of Florida Health Jacksonville, Jacksonville, USA; 2 Cardiology, University of Florida Health Jacksonville, Jacksonville, USA

**Keywords:** heerfordt-waldenström syndrome, uveoparotid fever, sarcoidosis, cardiac sarcoidosis

## Abstract

Sarcoidosis is a granulomatous disease histologically characterized by non-caseating granulomas. Although it usually affects the lungs, it can affect any organ system and present with a wide variety of symptoms. Heerfordt-Waldenström Syndrome, or uveoparotid fever, is a rare form of sarcoidosis that presents with a combination of fever, parotitis, facial paralysis, and uveitis. In this case report, we demonstrate a rare manifestation of sarcoidosis in a patient who presents with both the aforementioned syndrome and cardiac involvement. This case serves to highlight the importance of identifying the various clinical manifestations and management of systemic sarcoidosis.

## Introduction

Sarcoidosis is a multi-organ inflammatory disease of unknown etiology. It is characterized by the deposition of non-caseating granulomas in various tissues, and patients are generally diagnosed early in adulthood, usually before the age of 50. The incidence and prevalence of sarcoidosis are highest in African Americans. The disease can affect multiple organ systems: musculoskeletal, respiratory, ocular, cardiovascular, gastrointestinal, lymphatic, and neurological systems [1]. Heerfordt-Waldenström syndrome is a rare presentation of sarcoidosis. First described by Dr. Christian Heerfordt in the early 1900s, and later connected to sarcoidosis by Dr. Waldenström, the disease presents with enlargement of the parotid glands, facial nerve palsy, anterior uveitis, and fever [2, 3]. It is reported in the literature to occur in about 0.3% of sarcoidosis cases [4]. A slightly more common manifestation of sarcoidosis includes cardiac involvement. Cardiac sarcoidosis is diagnosed clinically in about five percent of sarcoidosis cases [5]. It can affect any portion of the heart and present as heart failure, sudden cardiac death, new arrhythmias, or unexplained heart block [6]. This case discusses an African American male who presents with both Heerfordt-Waldenström syndrome and cardiac involvement. 

## Case presentation

**Figure 1:**
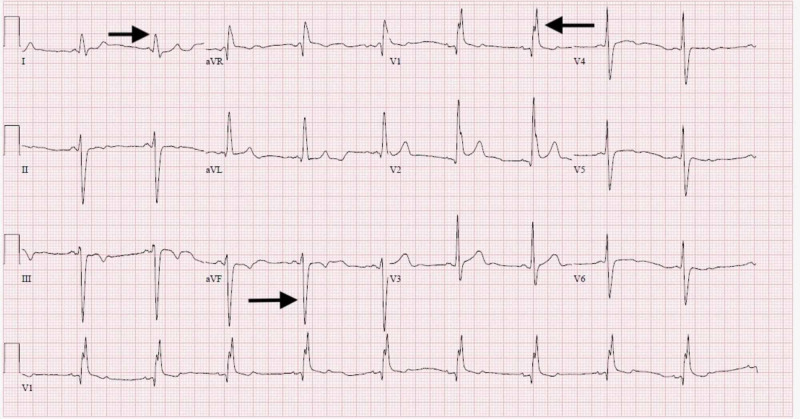
EKG with bifascicular block Arrows on the left showing left axis deviation and findings consistent with a left anterior fascicular block, while the arrow on the right demonstrating right bundle branch block.

**Figure 2:**
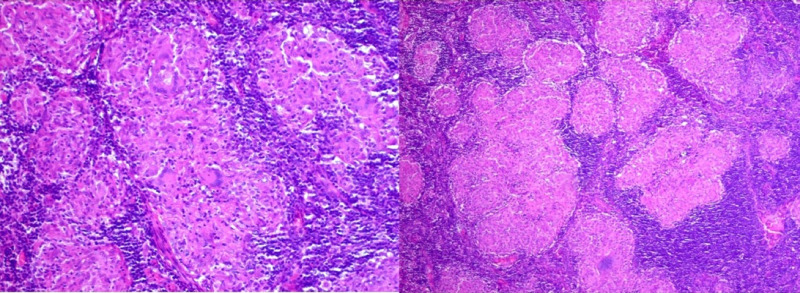
Submandibular lymph node biopsy with characteristic sarcoid granuloma

A 49-year-old African American male presented to the cardiology outpatient clinic for evaluation of recurrent syncope and palpitations. He had a one-month history of intermittent fevers with associated dry mouth and eyes. His physical examination revealed a bilateral parotid gland enlargement, cervical adenopathy, and facial nerve palsy, with eye redness. The patient's medical history was notable for longstanding hypertension. He had no known cardiac disease or neurological disorder. The initial electrocardiogram showed sinus rhythm with bifascicular block (Figure 1). Brain natriuretic peptide (BNP) level was slightly elevated at 250 (pg/mL). A transthoracic echocardiogram revealed septal and apical hypokinesis with preserved systolic function. An event monitor was ordered, and revealed symptom correlation with sinus pauses. Serology for tuberculosis, human immunodeficiency virus, antinuclear, anti‐Ro, and anti‐La antibodies were all negative. Other causes for heart block, such as Lyme disease, were considered but excluded based on geographic distribution. A chest radiograph was performed and negative for hilar adenopathy, infiltrates, or fibrosis. A submandibular lymph node biopsy was obtained, revealing non-caseating granulomas consistent with systemic sarcoidosis (Figure 2).

**Figure 3:**
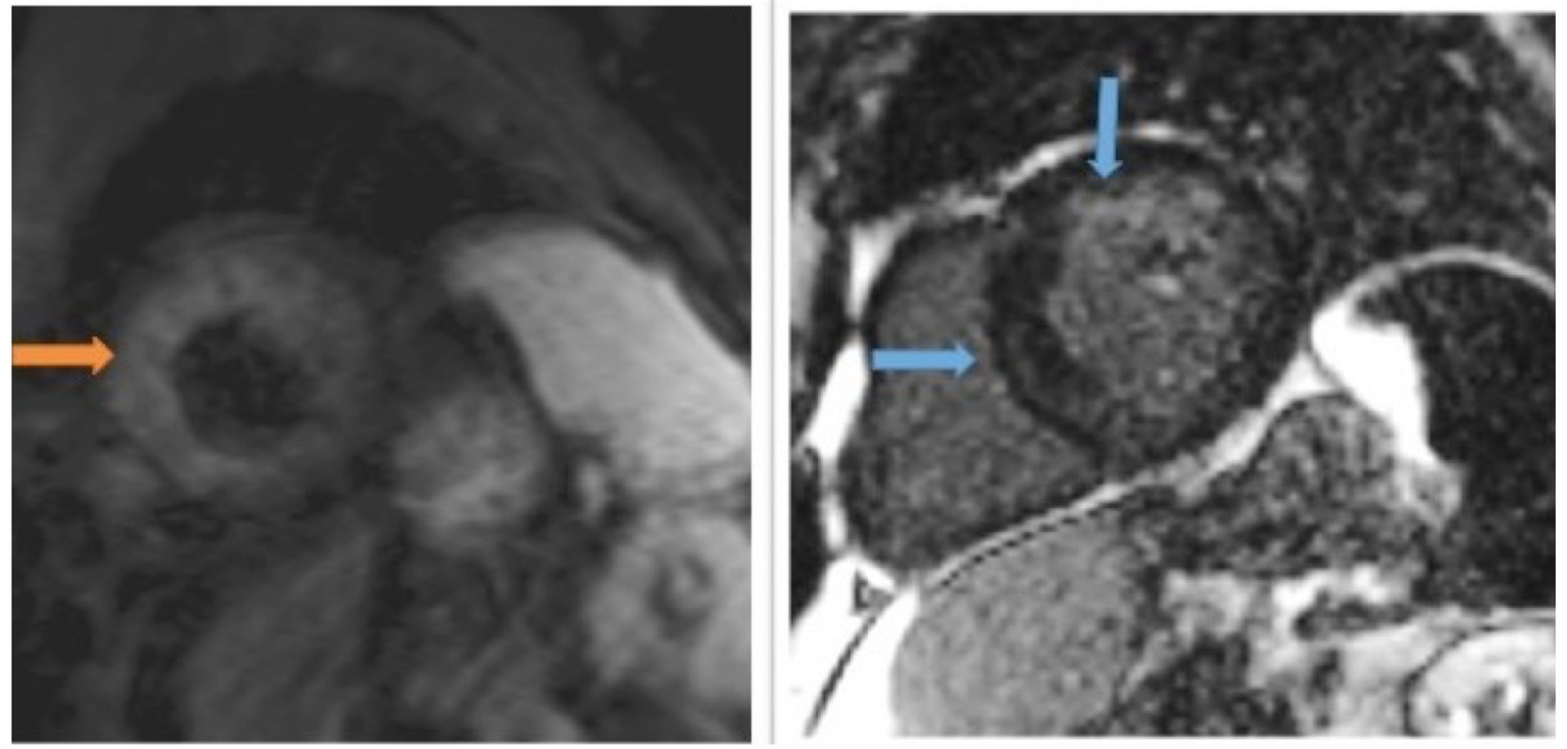
Short axis triple inversion sequence (left image) and short axis T1 delayed enhancement with gadolinium (right image) The left image shows edema in the mid myocardium (orange arrow). The right image shows patchy enhancement within the mid myocardium (blue arrows) indicative of fibrosis.

**Figure 4:**
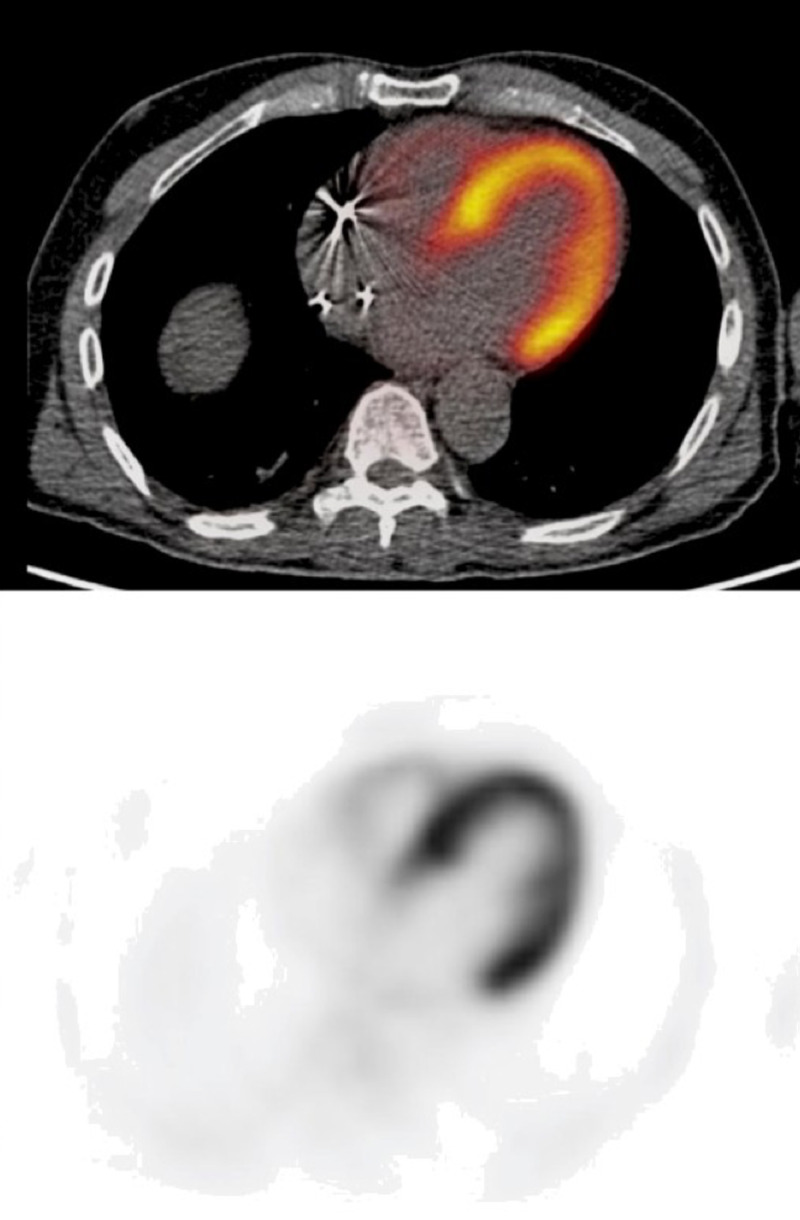
18F-FDG PET scan revealing normal perfusion at baseline without abnormal uptake suggesting resolution of cardiac sarcoid involvement 18F-FDG - 18F-fluorodeoxyglucose

On follow up cardiac magnetic resonance imaging (CMR), patchy late gadolinium enhancement (LGE) was appreciated in the septal wall and apical region concerning for cardiac sarcoidosis (Figure 3). The patient was subsequently diagnosed with cardiac sarcoidosis, along with Heerfordt-Waldenström syndrome. He was initially started on a high dose course of oral corticosteroids with a gradual taper over one year. Due to the frequent sinus pauses with correlation to the patient's syncopal episodes, a dual-chamber pacemaker was implanted. Upon a six-month follow-up, a cardiac 18F-fluorodeoxyglucose (18F-FDG) PET scan was ordered and showed resolution of cardiac involvement (Figure 4). On follow up appointment, a pacemaker device interrogation showed a minimal need for pacing. He reported improvement in his symptoms, and physical exam had complete resolution of uveitis, parotitis, and facial nerve palsy.

## Discussion

Heerfordt-Waldenström syndrome is a rare presentation of sarcoidosis characterized by enlargement of the parotid glands, facial nerve palsy, anterior uveitis, and fever. The clinical presentation varies from asymptomatic disease to acute illness, to chronic insidious organ failure. Facial nerve palsy is an integral component with an incidence of 25-50% in this syndrome. Parotid gland involvement occurs in about six percent and is usually bilateral. The majority have a low-grade fever. The involvement of the eye occurs in about 11-83% of patients, most commonly with anterior uveitis, along with increased lacrimation, photophobia, and blurred vision [2].

The diagnosis of Heerfordt-Waldenström syndrome is made by the clinical presentation along with a lymph node biopsy revealing non-caseating granulomas. When all symptoms are present, it is considered the complete type; when two of three symptoms occur (facial nerve palsy, enlarged parotid glands, anterior uveitis), it is considered incomplete type [7].

Cardiac sarcoidosis (CS) can affect any portion of the heart including the pericardium, atria, ventricles, papillary muscles, valves, and coronary arteries. Cardiovascular manifestations include congestive heart failure (25-75%), sudden cardiac death (25-65%), complete heart block (23-30%), bundle branch block (12-32%), and ventricular tachycardia 23%. Other manifestations include pericardial effusions, pulmonary hypertension, and ventricular aneurysms. Complete heart block is one of the most common findings, and patients are generally younger when it occurs with sarcoidosis vs. other etiologies. Clinically CS may be suspected in patients presenting with new and unexplained heart block, atrial arrhythmias, ventricular arrhythmias, and left ventricular dysfunction [8].

To date, there are no universally accepted guidelines for the diagnosis of CS. Two guidelines exist, one is the Japanese Ministry of Health and Welfare’s set of Criteria (published in 1993 and revised in 2007). The second proposed is by the National Institutes of Health’s A Case Control Etiology of Sarcoidosis Study set of criteria (published in 1999 and updated in 2014 by the World Association of Sarcoidosis and Other Granulomatous Disorders [WASOG]). Taking these into consideration, the Heart Rhythm Society (HRS) Expert Consensus Recommendations on Criteria for the Diagnosis of Cardiac Sarcoidosis report two pathways to the diagnosis of cardiac sarcoidosis. The first involves the presence of non-caseating granuloma on histological examination of myocardial tissue with no alternative causes. The second pathway requires a histological diagnosis of extra-cardiac sarcoidosis along with one or more of the following: steroid-responsive cardiomyopathy or heart block, unexplained reduced left ventricular ejection fraction, unexplained sustained VT, high-grade heart block, patchy uptake on dedicated cardiac PET, late gadolinium enhancement on cardiac MR (CMR), and/or positive gallium uptake. It is important to note that other causes for the cardiac manifestations must have been excluded [1].

While endomyocardial biopsy plays a role in the diagnosis of CS, the procedure has a low sensitivity of diagnosis due to the focal nature of the disease. When there is extra-cardiac sarcoidosis, the lymph node or lung biopsy provides a higher diagnostic yield with lower adverse outcomes and is often the preferred method to obtain a tissue diagnosis [5].

Echocardiography is often the first imaging modality when suspecting CS. There are no pathognomonic findings, although nonspecific findings include regional wall motion abnormalities, dilated left ventricle, and impaired systolic or diastolic function. Right heart pressures can be elevated due to coexisting lung involvement. CMR is the next imaging modality when suspecting sarcoidosis with cardiac involvement. The main finding is the presence of late gadolinium enhancement (LGE), which indicates a component of fibrosis. The typical LGE pattern in a cardiac sarcoidosis patient involves the sub-epicardial and mid-wall LGE along the basal septum and/or inferolateral wall. CMR can also detect right-sided ventricular dysfunction, which can be due to elevated right heart pressures from pulmonary sarcoidosis or right ventricular granulomatous infiltration. Nuclear imaging with 18F-FDG PET has emerged as an important diagnostic imaging modality in CS. The imaging interpretation requires combining a resting perfusion scan and an 18F-FDG PET scan. The findings can be described: normal, early-stage, progressive disease, and fibrous disease [5]. In addition to diagnosing CS, 18F-FDG PET has been shown to be beneficial to assess response to corticosteroids [9].

The treatment of systemic sarcoidosis most commonly has been corticosteroid therapy. The optimal dose of corticosteroid therapy for CS or Heerfordt-Waldenström syndrome is not known. Generally, the starting dose is 60 mg/day of prednisone, with a gradual reduction to a maintenance level of 10 to 15 mg/day over one year. Corticosteroid treatment will usually lead to the resolution of facial nerve palsy, uveitis, and parotid gland swelling [2]. Alternative agents such as methotrexate, azathioprine, infliximab, or mycophenolate mofetil may be given to patients who do not respond to corticosteroids or who are unable to tolerate steroids [5]. It should be pointed out that these agents are generally added in cases refractory to steroids and are not started initially. In the case presented, our patient responded successfully to prednisone and, therefore, did not require further immunosuppressive treatment. In regards to Heerfordt-Waldenström syndrome, artificial tears may also be implemented when facial paralysis with lagophthalmos is present [2]. 

Since sudden death may be the first sign of cardiac sarcoidosis, electrophysiological studies to detect any conduction delays or increased risk of sustained arrhythmias should be strongly considered in patients with suspected cardiac sarcoidosis. Pacemakers are placed for complete heart block, and an implantable cardioverter-defibrillator (ICD) is placed for ventricular fibrillation or tachycardia and markedly reduced ejection fraction [1].

## Conclusions

This clinical case illustrates that despite the various manifestations of systemic sarcoidosis, early diagnosis using a multimodality approach can facilitate early treatment and symptom resolution. The systemic manifestations of sarcoidosis are numerous, and the diagnosis of both CS and Heerfordt-Waldenström syndrome require a high degree of clinical suspicion. Heerfordt-Waldenström syndrome was diagnosed in our patient based on clinical symptoms including fevers, dry eyes and mouth, bilateral parotid gland enlargement, cervical adenopathy, and facial nerve palsy. Per the HRS expert Consensus Statement, our patient met the criteria for CS based on a histological diagnosis of extra-cardiac sarcoidosis (submandibular lymph node biopsy) along with late gadolinium enhancement on CMR. Both manifestations revealed a very favorable treatment response to a gradual corticosteroid taper. To date, there are few case reports described in the literature of Heerfordt-Waldenström Syndrome with concomitant cardiac sarcoidosis. The association and implications of cardiac sarcoidosis with other types of sarcoid requires further investigations. Additionally, testing for treatment response with post treatment imaging has not been well established, but may a reasonable option to monitor response and disease activity.
